# The experience of life events and body composition in middle childhood: a population-based study

**DOI:** 10.1186/s12966-021-01188-7

**Published:** 2021-08-25

**Authors:** Ivonne P. M. Derks, Sara Hannani, Florianne O. L. Vehmeijer, Henning Tiemeier, Pauline W. Jansen

**Affiliations:** 1 Department of Psychology, Education, and Child Studies, Erasmus University Rotterdam, Burgemeester Oudlaan 50, 3062 PA Rotterdam, the Netherlands; 2 Department of Child and Adolescent Psychiatry/ Psychology, Erasmus MC-University Medical Center, Rotterdam, the Netherlands; 3 The Generation R Study Group, Erasmus MC-University Medical Center, Rotterdam, the Netherlands; 4 Department of Pediatrics, Erasmus MC-University Medical Center, Rotterdam, the Netherlands; 5 Department of Social and Behavioral Sciences, Harvard TH Chan School of Public Health, Boston, MA USA

**Keywords:** Adverse childhood experiences, Adversity, Childhood obesity, Adiposity, Body composition, BMI

## Abstract

While studies suggest potential influences of childhood adversities on obesity development in adulthood, less is known about the short-term association in children. We examined the association between a wide range of life events experienced in the first ten years of life (including maltreatment and milder adversities) and body composition in 5333 ten-year old Dutch children. In structured interviews, mothers retrospectively reported on their children’s experience of 24 events. BMI was calculated, and fat mass index and fat free mass index were determined by dual-x-ray absorptiometry scanning. Linear regressions showed that, unadjusted, a higher number of life events was associated with higher BMI and body composition. However, associations attenuated to non-significance after adjustment for covariates. Similar findings were observed for maltreatment and milder life events. Thus, the number of experienced life events was not associated with body composition in middle childhood. Rather, other factors, like socioeconomic conditions, accounted for the relationship between life events and weight development in children.

**Supplementary Information:**

The online version contains supplementary material available at 10.1186/s12966-021-01188-7.

## Introduction

The experience of adversity in childhood can have lasting effects on people’s health and well-being throughout the life course [1, 2]. In this context, it is shown that adults who experienced adversity during childhood have a heightened risk of becoming overweight or obese [3, 4]. Proposed mechanisms for the relationship between early-life adversity and obesity in adults are mostly related to physical stress responses, DNA methylation in genes associated with obesity risk, disturbed emotion regulation, increased appetite and a tendency towards (emotional) overeating [5–7]. However, it is unclear whether exposure to adversities already affects weight gain in childhood. Two systematic reviews including studies in children and adolescents reported opposing findings [3, 8], and more recent studies continued to report inconsistent findings [9–14]. Most of these studies focused solely on maltreatment (including physical, emotional and sexual abuse or neglect) or other severe adversities such as parental incarceration or domestic violence [3, 9–11, 14], while milder and more common life events, which may also induce the experience of stress, such as peer problems, changing schools, and parental conflict, were often not considered. Finally, only one study examined Fat Mass Index (FMI) and android fat percentage as indicators of adiposity in adolescents and observed no association with lifetime exposure to adversity [13]. In this population-based study, we examined the association between the number of experienced life events in the first ten years of life and body composition in 5333 ten-year-old children.

## Methods

This study was embedded in The Generation R Study, a multi-ethnic, population-based cohort in Rotterdam, the Netherlands [15]. The Medical Ethical Committee of the Erasmus Medical Center approved the study. Consent for the examination at age 10 years was obtained from parents of 7393 children, of whom 5862 children visited the research center. The final study population consisted of 5333 children with information on life events and body composition.

Mothers were interviewed by trained research assistants about their offspring’s experience of life events. This retrospective, structured interview included 24 events. This interview was similar to a previous assessment in TRAILS, another Dutch cohort of young adolescents [16]. TRAILS based the selection of events on life events in previous questionnaires, including the Life Event Checklist [17], and subsequently adapted and added events to match the age of the participants, cohort design and cohort characteristics, as many life events that are assessed in validated questionnaires [18, 19] were not applicable for young adolescents. Examples of events in this interview are parental separation, conflicts, illness, transferring schools and sexual and physical abuse. The total number of experienced life events was calculated, ranging from 0 to 24. Life events that were possibly related to each other (such as divorce, financial problems and conflict within the family) were counted as separate events. Multiple occurrences were not taken into account, but if this applied, mothers were asked to report on the most severe event. In addition to the total number of events, a separate score was created for the experience of physical abuse, sexual abuse or threat (maltreatment) versus milder life evens (all events except for maltreatment). The total number of events and of milder events were included in the analyses as continuous variables, while maltreatment was studied as a binary variable given the highly skewed distribution. In Additional Table 1, the prevalence of each event in the sample is reported.

Child height was measured using a Harpenden stadiometer and weight was measured using a mechanical personal scale (SECA), without shoes and heavy clothing. From this, BMI was calculated as well as sex- and age-adjusted Body Mass Index (BMI) SD scores according to Dutch reference growth curves [20]. Further, body fat mass and fat free mass were assessed by Dual-energy-X-ray-Absorptiometry scanning (iDXA, GE-LUNAR, 2009, Madison, WI, USA). Sex- and age-adjusted FMI and Fat Free Mass Index (FFMI) SD scores were calculated based on all available data at age ten years.

Based on previous literature, potential confounders were considered. During pregnancy, mothers and fathers each filled out questionnaires including assessments of: country of birth, from which child ethnicity was derived; highest level of obtained education of both parents; household income; and parental psychiatric symptoms as assessed with the validated Brief Symptom Inventory [21]. Fathers reported their weight and height by questionnaire, from which paternal BMI was calculated. During early pregnancy, mothers’ height and weight were measured by research staff, from which maternal BMI was calculated. Children’s birth weight and sex were derived from medical records.

Differences in sample characteristics by the number of life events were examined with Chi Square tests, one-way ANOVAs or Kruskall-Wallis tests. Furthermore, correlations between the number of life events, body composition and covariates were examined with Pearson, Spearman Rank and Cramer’s V statistics. The relationship between life events and body composition at ten years was examined with multivariable linear regression analyses (separate analyses for BMI, BMI SD score, FMI SD score and FFMI SD score). Two models were created: the first model was unadjusted (for BMI, sex- and age adjusted), and a second model adjusted for all covariates described above except for paternal BMI, which did not influence the association by more than 5%. These models were rerun to test the relation of maltreatment or milder events with body composition. Three sensitivity analyses were conducted: analyses were repeated 1) excluding events related to socioeconomic conditions, 2) in which we only included events that had a moderate or high negative perceived impact at the time of the interview according to the mother and 3) in which only events experienced before the age of 6 years was studied in relation to the change (delta) in body composition from 6 to 10 years. Missings on covariates were estimated with multiple imputation and pooled results from 20 datasets are presented. All analyses were performed with IBM SPSS Statistics 25.

## Results

Sample characteristics are presented in Table 1. Mothers reported that on average, children experienced 4.3 life events (SD = 2.6) until the age of 10 years. Children who experienced 6 or more events (27%) were more likely to have a Non-Western background and a low household income than children who experienced 0–2 life events. Moreover, parents of children with more life events also reported more psychiatric symptoms and mothers had a higher BMI, while no differences in paternal BMI were observed. Correlations between the number of life events, body composition and covariates are presented in Additional Table 2.

**Table 1:** Sample characteristics by number of life events experienced during childhood

		**Number of life events (0–10 years)**			
**Child characteristics**	**Total** **(*n* = 5333)**	**0–2 events** **(*n* = 1443)**	**3–5 events** **(*n* = 2438)**	**6 or more events** **(*n* = 1452)**	***p*-value**
Age at 10 years visit, mean (SD)	9.78 (0.33)	9.75 (0.30)	9.78 (0.32)	9.83 (0.39)	< 0.001
Sex, % girl	50.8	51.1	50.9	50.3	0.91
Ethnicity, % Dutch	61.6	62.7	63.5	57.2	0.001
% Other western	9.0	9.5	8.7	9.0	
% Non-western	29.4	27.8	27.8	33.8	
Birth weight for gestational age SD score	-0.07 (1.02)	-0.04 (0.99)	-0.05 (1.01)	-0.14 (1.06)	0.009
BMI at 10 years visit, mean (SD)	17.54 (2.73)	17.32 (2.63)	17.49 (2.63)	17.85 (2.97)	< 0.001
FMI at 10 years visit, mean (SD)	4.85 (2.07)	4.71 (2.00)	4.80 (2.00)	5.07 (2.23)	< 0.001
FFMI at 10 years visit, mean (SD)	12.59 (1.09)	12.52 (1.06)	12.59 (1.06)	12.66 (1.16)	0.002
**Family characteristics**					
Maternal educational level, % low	18.4	15.2	16.2	25.4	< 0.001
% medium	30.6	26.4	28.8	37.8	
% high	51.0	58.4	55.0	36.8	
Paternal educational level, % low	18.7	16.0	15.8	26.8	< 0.001
% medium	26.6	23.5	26.0	31.0	
% high	54.8	60.4	58.2	42.2	
Household income, % low (< 1600 per month)	13.8	8.9	11.9	22.6	< 0.001
% medium (1600–4000 per month)	50.1	48.2	48.2	55.6	
% high (> 4000 per month)	36.1	42.9	40.0	21.8	
Maternal BMI, median [IQR]	23.71 [4.85]	23.38 [4.49]	23.67 [4.54]	24.03 [5.28]	< 0.001
Paternal BMI, median [IQR]	24.93 [4.17]	24.93 [4.29]	24.92 [4.05]	25.00 [4.31]	0.674
Maternal psychiatric symptoms (prenatal assessment), median [IQR]	0.15 [0.25]	0.12 [0.19]	0.14 [0.22]	0.21 [0.37]	< 0.001
Paternal psychiatric symptoms (prenatal assessment), median [IQR]	0.06 [0.15]	0.05 [0.14]	0.06 [0.13]	1.00 [0.19]	< 0.001

Based on original data

Table 2 presents the association between the number of life events and body composition. The number of experienced events was positively associated with BMI, BMI SD score, FMI SD score and FFMI SD score (for instance, FMI SD score: B per event = 0.03, 95% CI = 0.02, 0.04) in children. However, after adjustment for confounders, the associations attenuated to statistical non-significance. Maternal education and psychopathology symptoms, as well as household income mostly accounted for this attenuation (not tabulated). Similar results were observed for mild life events and maltreatment as separate predictors. Sensitivity analyses (1. without socioeconomic related life events; 2. after excluding life events with a low perceived impact; 3. after only including life events prior to the age of 6 years with the change in body composition from 6 to 10 years) also indicated similar patterns of associations (Additional Tables 3–5).

**Table 2:** Associations between the number of life events experienced during childhood and body composition at ten years

			Body composition at age 10 years			
			BMI	BMI SD score	FMI SD score	FFMI SD score
			B (95% CI)	B (95% CI)	B (95% CI)	B (95% CI)
Total life events	Model 1^a^	Per event	0.08 (0.05, 0.11)	0.03 (0.02, 0.04)	0.03 (0.02, 0.04)	0.01 (0.00, 0.02)
	Model 2^b^	Per event	0.01 (-0.01, 0.04)	0.01 (-0.00, 0.02)	0.00 (-0.01, 0.01)	0.01 (-0.00, 0.02)
Events divided by severity						
Milder life events	Model 1^a^	Per event	0.08 (0.05, 0.11)	0.03 (0.02, 0.04)	0.03 (0.02, 0.04)	0.02 (0.00, 0.03)
	Model 2^b^	Per event	0.01 (-0.02, 0.04)	0.01 (-0.00, 0.02)	-0.00 (-0.01, 0.01)	0.01 (-0.00, 0.02)
Maltreatment^**c**^		0 events	Reference	Reference	Reference	Reference
	Model 1^a^	1 or more events	0.26 (0.07, 0.44)	0.12 (0.05, 0.19)	0.11 (0.04, 0.17)	0.04 (-0.03, 0.10)
	Model 2^b^	1 or more events	0.04 (-0.13, 0.21)	0.04 (-0.02, 0.11)	0.01 (-0.05, 0.07)	0.00 (-0.06, 0.07)

*N* = 5333 (*n* = 1047 with ≥ 1 maltreatment event)

^a^ Unadjusted (for BMI, adjusted for sex and age at outcome)

^b^ Additionally adjusted for child ethnicity and birth weight, household income and maternal and paternal education, maternal BMI and maternal and paternal psychopathology symptoms

^c^ Maltreatment included events on physical threat or abuse and sexual threat or abuse

## Discussion

Results of this population-based study using retrospective information on life events, suggest that the amount of events children experienced in the first 10 years of life was not associated with BMI, FMI or FFMI at the age of 10 years. Our findings indicate that the relationship between life events and obesity development in children was accounted for by other factors, of which parental socioeconomic indicators and psychopathological symptoms of the mother during pregnancy were most important.

Our findings are in line with several other studies that reported mostly no association between the experience of severe adversity and weight status in childhood or adolescence [3, 9, 13]. This incongruence with the robust findings in adults might entail that weight-related consequences of stressful events during childhood appear only later in life [3]. The long-term associations in adults are potentially explained by underlying biological and behavioral mechanisms that are activated during childhood or adolescence, including elevated inflammation and cortisol or emotional eating, that may have a cumulative effect that only becomes visible years later [6, 22].

Results showed that the association between life events and body composition was explained by other factors, including socioeconomic conditions and maternal psychopathology. Some of the events included in this study, namely financial difficulties, neighborhood problems or involuntarily unemployment, might be strongly associated with socioeconomic conditions of the family. Yet, the sensitivity analysis in which these socioeconomic-related events were removed from the total events resulted in similar findings. In line with our findings, two population-based studies on early-life abuse and adolescent obesity risk also reported that the relationship was accounted for by other factors, including socioeconomic status [9, 13]. For non-abusive adversity, it was suggested that interventions focusing on improving socioeconomic conditions may protect against obesity [23].

Strengths of this study were the assessment of a broad range of events, the assessment of body composition and a large sample size. However, although the life events interview was similar to a previous assessment in another cohort, this interview was not validated. Moreover, in our analyses, different adverse events were equally weighted, while it can be hypothesized that one event might affect a child more than other events. Likewise, lasting or repeated events might also be more harmful as compared to single occurrences. Yet, (the tendency towards) physical and sexual abuse are highly stressful events that were not associated with body composition. Furthermore, the sensitivity analysis in which only events with a moderate or high perceived impact were included also showed similar null findings after careful adjustment. Finally, this study relied on a mother-report of adversity which could have resulted in socially desirable answers. Although the interviewers were trained to create a trustful environment, some mothers might have avoided to confirm the occurrence of certain events resulting in an underreport of events.

In conclusion, in this population-based cross-sectional study of ten-year-old children, no association between the experience of life events and body composition was observed. This suggests that other factors than the experience of life events explain the obesity epidemic in children.

## Supplementary Information

**Additional file 1: Additional Table 1.** Frequency of life events in the study sample at the age of ten years (*n*=5333). **Additional Table 2.** Correlations between the number of life events, body composition and covariates. **Additional Table 3.** Associations between the number of life events, without socioeconomic-related life events, during childhood and body composition at ten years. **Additional Table 4.** Associations between the number of life events rated as influential by mothers and body composition at ten years. **Additional Table 5.** Associations between the number of life events from 0 to 5 years and the change in body composition from 6 to 10 years.

## Data Availability

The data that support the findings of this study are available from data management Generation R but restrictions apply to the availability of these data, which were used under license for the current study, and are therefore not publicly available. Data are, however, available from the authors upon reasonable request and with permission of Vincent Jaddoe. Interested researchers may contact Vincent Jaddoe (v.jaddoe@erasmusmc.nl).

## Abbreviations

BMI: Body Mass Index
CI: Confidence Interval
FMI: Fat Mass Index
FFMI: Fat Free Mass Index
SD: Standard deviation
