# Supplementary material for: The experience of life events and body composition in middle childhood: a population-based study
Source: Int J Behav Nutr Phys Act. 2021 Aug 25;18:109. doi: 10.1186/s12966-021-01188-7 (PMC8386051; doi:10.1186/s12966-021-01188-7)
Supplement: Supplementary file 1 — Additional file 1: Additional Table 1. Frequency of life events in the study sample at the age of ten years (n=5333). Additional Table 2. Correlations between the number of life events, body composition and covariates. Additional Table 3. Associations between the number of life events, without socioeconomic-related life events, during childhood and body composition at ten years. Additional Table 4. Associations between the number of life events rated as influential by mothers and body composition at ten years. Additional Table 5. Associations between the number of life events from 0 to 5 years and the change in body composition from 6 to 10 years. [file 12966_2021_1188_MOESM1_ESM.docx]

| **Additional Table 1. Frequency of life events in the study sample at the age of ten years (n=5333)** | | | |
| --- | --- | --- | --- |
|  | Item | Category^1^ | Frequency (%) |
| 1 | Did your child get seriously sick or did he/she have an accident? | Milder event | 1509 (28.3) |
| 2 | Did a family member get seriously sick or did someone have a serious accident? | Milder event | 1223 (22.9) |
| 3 | Did someone else, who is important to the child, get seriously sick or did someone have a serious accident? | Milder event | 1786 (33.5) |
| 4 | Has the father/mother or other caretaker of your child died? | Milder event | 40 (0.8) |
| 5 | Has someone else, who your child cared a lot about, passed away? | Milder event | 1906 (35.7) |
| 6 | Has a pet, who you child cared a lot about, die? | Milder event | 1647 (30.9) |
| 7 | Does or did your child have to deal with a high workload at school? | Milder event | 1403 (26.3) |
| 8 | Has your child ever repeated a grade? | Milder event | 577 (10.8) |
| 9 | Are/were there any neighbourhood problems? E.g. vandalism or insecurity. | Milder event, socioeconomic event | 941 (17.6) |
| 10 | Has your family financial difficulties or had your family ever have them? | Milder event, socioeconomic event | 796 (14.9) |
| 11 | Does your child have ongoing conflicts with a family member (or did your child ever have them)? | Milder event | 230 (4.3) |
| 12 | Does your child have ongoing conflicts with someone else (or did your child ever have them)? | Milder event | 463 (8.7) |
| 13 | Do other family member have ongoing conflicts with each other (or did they ever have them)? | Milder event | 593 (11.1) |
| 14 | Are you and your partner divorced or separated? | Milder event | 1157 (21.7) |
| 15 | Did one of the parents become involuntarily unemployed? | Milder event, socioeconomic event | 1408 (26.4) |
| 16 | Did your child lose a good friend due to an argument? | Milder event | 188 (3.5) |
| 17 | Did your child ever lose something which was important to him/her? E.g. through fire, loss, or theft. | Milder event | 605 (11.3) |
| 18 | Has someone ever used physical violence against your child? For example, beating him/her up. | Maltreatment | 383 (7.2) |
| 19 | Has someone almost used physical violence against your child? So that it not actually happened, but your child was frightened. | Maltreatment | 633 (11.9) |
| 20 | Has someone made sexual comments or movements towards your child? | Maltreatment | 194 (3.6) |
| 21 | Has your child experienced inappropriate sexual behaviour? | Maltreatment | 82 (1.5) |
| 22 | Has someone spread mean rumours about your child? | Milder event | 470 (8.8) |
| 23 | Has your child moved to a different place of residence? | Milder event | 3154 (59.1) |
| 24 | Has your child changed schools? | Milder event | 1425 (26.7) |

| **Additional Table 2. Correlations between the number of life events, body composition and covariates.** | | | | | | | | | | | | | | | | | |
| --- | --- | --- | --- | --- | --- | --- | --- | --- | --- | --- | --- | --- | --- | --- | --- | --- | --- |
|  | 1. | 2. | 3. | 4. | 5. | 6. | 7. | 8. | 9. | 10. | 11. | 12. | 13. | 14. | 15. | 16. | 17. |
| 1. Age at 10 years visit | 1 |  |  |  |  |  |  |  |  |  |  |  |  |  |  |  |  |
| 1. Sex (girl) | -0.01 | 1 |  |  |  |  |  |  |  |  |  |  |  |  |  |  |  |
| 1. Ethnicity (Dutch) | -0.09* | 0.01 | 1 |  |  |  |  |  |  |  |  |  |  |  |  |  |  |
| 1. Ethnicity (Other Western) | -0.01 | 0.02 | 0.00 | 1 |  |  |  |  |  |  |  |  |  |  |  |  |  |
| 1. Ethnicity (Non-western) | 0.10 | 0.02 | 0.00 | 0.00 | 1 |  |  |  |  |  |  |  |  |  |  |  |  |
| 1. Birth weight SD score | -0.02 | -0.01 | 0.13* | -0.00 | -0.13* | 1 |  |  |  |  |  |  |  |  |  |  |  |
| 1. Total number of life events | 0.12* | -0.02 | -0.04* | -0.01 | 0.05 | -0.05* | 1 |  |  |  |  |  |  |  |  |  |  |
| 1. BMI SD score | 0.08* | -0.04* | -0.18* | 0.01 | 0.19* | 0.12* | 0.08* | 1 |  |  |  |  |  |  |  |  |  |
| 1. FMI SD score | -0.00 | -0.00 | -0.20* | 0.01 | 0.20* | 0.03* | 0.08* | 0.87* | 1 |  |  |  |  |  |  |  |  |
| 1. FFMI SD score | -0.00 | -0.02 | -0.08* | 0.01 | 0.08 | 0.19* | 0.04* | 0.74* | 0.46* | 1 |  |  |  |  |  |  |  |
| 1. Maternal educational level | -0.02 | -0.02 | 0.28* | 0.09* | -0.35* | 0.11* | -0.17* | -0.21* | -0.24* | -0.07* | 1 |  |  |  |  |  |  |
| 1. Paternal educational level | -0.03 | -0.02 | 0.17* | 0.04* | -0.27* | 0.12* | -0.15* | -0.16* | -0.20* | -0.03 | 0.52* | 1 |  |  |  |  |  |
| 1. Household income | -0.01 | 0.01 | 0.33* | 0.02 | -0.37* | 0.11* | -0.20* | -0.16* | -0.20* | -0.04* | 0.50* | 0.45* | 1 |  |  |  |  |
| 1. Maternal BMI | 0.05* | -0.00 | -0.09* | -0.10* | 0.16* | 0.15* | 0.07* | 0.34* | 0.33* | 0.22* | -0.22* | -0.16* | -0.16* | 1 |  |  |  |
| 1. Paternal BMI | 0.00 | 0.01 | -0.06* | -0.00 | 0.07* | 0.04* | 0.01 | 0.27* | 0.25* | 0.19* | -0.12* | -0.12* | -0.03 | 0.21* | 1 |  |  |
| 1. Maternal psychiatric symptoms | 0.05* | -0.02 | -0.25* | 0.02 | 0.25* | -0.07* | 0.23* | 0.13* | 0.14* | 0.06* | -0.24* | -0.18* | -0.30* | 0.09* | 0.00 | 1 |  |
| 1. Paternal psychiatric symptoms | 0.02 | -0.00 | -0.16* | 0.04* | 0.16* | -0.07* | 0.14* | 0.06* | 0.08* | 0.02 | -0.10* | -0.11* | -0.23* | 0.03 | -0.02 | 0.27* | 1 |
| Based on original data. Values represent Pearson-, Spearman Rank- or Cramer’sV correlation coefficients.  *=p value ≤0.05 | | | | | | | | | | | | | | | | | |

| **Additional Table 3. Associations between the number of life events, without socioeconomic-related life events, during childhood and body composition at ten years.** | | | | | | |
| --- | --- | --- | --- | --- | --- | --- |
|  |  |  | **Body composition at age 10 years** | | | |
|  |  |  | **BMI** | **BMI SD score** | **FMI SD score** | **FFMI SD score** |
|  |  |  | **B (95% CI)** | **B (95% CI)** | **B (95% CI)** | **B (95% CI)** |
| **Total life events** | Model 1^1^ | Per event | 0.07 (0.03, 0.10) | 0.03 (0.02, 0.04) | 0.02 (0.01, 0.04) | 0.01 (-0.00, 0.02) |
|  | Model 2^2^ | Per event | 0.02 (-0.01, 0.05) | 0.01 (-0.00, 0.02) | 0.00 (-0.01, 0.01) | 0.01 (-0.01, 0.02) |
|  |  |  |  |  |  |  |
| **Events divided by severity^3^** | |  |  |  |  |  |
| **Milder life events** | Model 1^1^ | Per event | 0.07 (0.03, 0.10) | 0.03 (0.01, 0.04) | 0.02 (0.01, 0.04) | 0.01 (-0.00, 0.02) |
|  | Model 2^2^ | Per event | 0.02 (-0.02, 0.05) | 0.01 (-0.01, 0.02) | -0.00 (-0.01, 0.01) | 0.01 (-0.01, 0.02) |
| N=5333  ^1^ Unadjusted (for BMI, adjusted for sex and age at outcome).  ^2^ Adjusted for child ethnicity and birth weight, household income and maternal and paternal education, maternal BMI and maternal and paternal psychopathology symptoms.  ^3^Results for maltreatment are not presented because maltreatment did not include events depending on socioeconomic conditions and therefore results did not change. | | | | | | |

| **Additional Table 4. Associations between the number of life events rated as influential by mothers and body composition at ten years.** | | | | | | |
| --- | --- | --- | --- | --- | --- | --- |
|  |  |  | **Body composition at age 10 years** | | | |
|  |  |  | **BMI** | **BMI SD score** | **FMI SD score** | **FFMI SD score** |
|  |  |  | **B (95% CI)** | **B (95% CI)** | **B (95% CI)** | **B (95% CI)** |
| **Total life events^1^** | Model 1^2^ | Per event | 0.13 (0.07, 0.20) | 0.05 (0.02, 0.07) | 0.05 (0.03, 0.08) | 0.02 (-0.00, 0.05) |
|  | Model 2^3^ | Per event | 0.04 (-0.02, 0.11) | 0.02 (-0.01, 0.04) | 0.02 (-0.01, 0.04) | 0.01 (-0.01, 0.03) |
|  |  |  |  |  |  |  |
| **Events divided by severity** | |  |  |  |  |  |
| **Milder life events^4^** | Model 1^2^ | Per event | 0.14 (0.07, 0.21) | 0.05 (0.02, 0.08) | 0.06 (0.03, 0.08) | 0.02 (-0.00, 0.05) |
|  | Model 2^3^ | Per event | 0.05 (-0.01, 0.12) | 0.02 (-0.01, 0.04) | 0.02 (-0.01, 0.04) | 0.01 (-0.01, 0.04) |
|  |  |  |  |  |  |  |
| **Maltreatment^5^** |  | 0 events | Reference | Reference | Reference | Reference |
|  | Model 1^2^ | 1 or more events | 0.47 (0.03, 0.90) | 0.18 (0.02, 0.35) | 0.19 (0.03, 0.35) | 0.07 (-0.09, 0.23) |
|  | Model 2^3^ | 1 or more events | 0.07 (-0.33, 0.47) | 0.05 (-0.11, 0.20) | 0.02 (-0.12, 0.17) | 0.02 (-0.14, 0.17) |
| ^1^ For total life events, n=5273.  ^2^ Unadjusted (for BMI, adjusted for sex and age at outcome).  ^3^ Adjusted for child ethnicity and birth weight, household income and maternal and paternal education, maternal BMI and maternal and paternal psychopathology symptoms.  ^4^ For mild life events, total n= 5278.  ^5^ For maltreatment, total n=5326 (n=152 with one or more maltreatment event). Maltreatment included events on physical threat or abuse and sexual threat or abuse. | | | | | | |

| **Additional Table 5. Associations between the number of life events from 0 to 5 years and the change in body composition from 6 to 10 years.** | | | | | | |
| --- | --- | --- | --- | --- | --- | --- |
|  |  |  | **Change in body composition (6-10 years)** | | | |
|  |  |  | **∆ BMI** | **∆ BMI SD score** | **∆ FMI SD score** | **∆ FFMI SD score** |
|  |  |  | **B (95% CI)** | **B (95% CI)** | **B (95% CI)** | **B (95% CI)** |
| **Total life events** | Model 1^1^ | Per event | 0.04 (0.01, 0.07) | 0.02 (0.00, 0.03) | 0.02 (0.01, 003) | 0.01 (-0.00, 0.02) |
|  | Model 2^2^ | Per event | 0.00 (-0.03, 0.03) | 0.01 (-0.01, 0.02) | 0.01 (-0.00, 0.02) | -0.00 (-0.01, 0.01) |
|  |  |  |  |  |  |  |
| **Events divided by severity** | |  |  |  |  |  |
| **Milder life events** | Model 1^1^ | Per event | 0.04 (0.00, 0.07) | 0.02 (0.00, 0.03) | 0.02 (0.01, 0.03) | 0.01 (-0.00, 0.02) |
|  | Model 2^2^ | Per event | -0.00 (-0.03, 0.03) | 0.01 (-0.01, 0.02) | 0.01 (-0.01, 0.02) | 0.00 (-0.01, 0.01) |
|  |  |  |  |  |  |  |
| **Maltreatment^3^** |  | 0 events | Reference | Reference | Reference | Reference |
|  | Model 1^1^ | 1 or more events | 0.25 (0.00, 0.49) | 0.06 (-0.03, 0.16) | 0.08 (-0.01, 0.17) | 0.03 (-0.06, 0.12) |
|  | Model 2^2^ | 1 or more events | 0.14 (-0.09, 0.37) | 0.03 (-0.06, 0.12) | 0.04 (-0.05, 0.13) | -0.01 (-0.09, 0.08) |
| N=5333 (n= 173 with ≥1 maltreatment event).  ^1^ Unadjusted (for BMI, adjusted for sex and age at outcome).  ^2^ Adjusted for child ethnicity and birth weight, household income and maternal and paternal education, maternal BMI and maternal and paternal psychopathology symptoms.  ^3^ Maltreatment included events on physical threat or abuse and sexual threat or abuse (n= 174 with ≥1 maltreatment event). | | | | | | |
